# Effects of tryptophan depletion on anxiety, a systematic review

**DOI:** 10.1038/s41398-021-01219-8

**Published:** 2021-02-11

**Authors:** Simone M. E. Schopman, Renske C. Bosman, Anna D. T. Muntingh, Anton J. L. M. van Balkom, Neeltje M. Batelaan

**Affiliations:** 1 Amsterdam UMC, Vrije Universiteit, Department of Psychiatry, Amsterdam Public Health institute, Amsterdam, The Netherlands; 2 GGZ inGeest Specialized Mental Health Care, Amsterdam, The Netherlands

**Keywords:** Psychiatric disorders, Predictive markers

## Abstract

Vulnerability markers for onset of anxiety disorders are scarce. In depression, patients at risk tend to respond with a negative mood to ‘acute tryptophan depletion’ (ATD), while healthy volunteers and current patients do not. The serotonergic system thus provides indications for vulnerability for depression. It is unknown whether ATD reveals vulnerability in anxiety too. This study systematically reviews the effects of ATD on anxiety and assesses whether challenging anxiety modifies the response. PubMed, Embase and PsychInfo were systematically searched up to April 2019 for studies in which (1) healthy volunteers or patients with a (remitted) anxiety disorder underwent ATD and (2) levels of anxiety were reported. In total, 21 studies were included. Studies conducted in healthy volunteers (*n* = 13), and patients with a remitted (*n* = 6) or current (panic, social or generalised) anxiety disorder (*n* = 4). Studies were mostly of poor quality and heterogeneous regarding population, challenge test used and outcome measures. ATD did not consistently affect anxiety in any of the groups. Moreover, a challenge test after ATD (*n* = 17 studies) did not consistently provoke anxiety in healthy volunteers or remitted patients. A 35% CO_2_ challenge did consistently increase anxiety in patients with a current panic disorder (PD). To conclude, this systematic review found no clear indications that ATD provokes anxiety in those at risk for anxiety disorders. Hence, unlike in depression, ATD does not indicate vulnerability to develop an anxiety disorder. Because included studies were heterogeneous and mostly of poor quality, there is an urgent need for high quality research in homogeneous samples.

## Introduction

Anxiety disorders are severe and disabling disorders and many patients have a poor course trajectory^1–4^. Although sociodemographic and clinical factors have been associated with developing anxiety disorders (e.g. gender, disability)^5,6^, their predictive validity is low. Studies of biological factors (e.g. hypothalamic–pituitary–adrenal axis functioning, inflammation markers) show inconclusive results^7–10^. Currently, vulnerability to develop an anxiety disorder cannot be revealed.

In depressive disorders, the serotonergic (or 5-hydroxytryptamine [5-HT]) system provides an indication for vulnerability. This neurotransmitter system is involved in the regulation of emotions and seems dysregulated in depressive disorders^11^. The serotonin system in depression has been investigated repeatedly by lowering serotonin levels with ATD^12^. In ATD, the availability of the essential amino acid tryptophan, the dietary precursor of serotonin, is decreased by means of a specifically designed amino acid drink. ATD is assumed to result in a decrease of the availability of serotonin in the central nervous system^13^ (see Methods for more details).

Responding to ATD with a lower mood seems to reveal vulnerability for developing a depressive episode. A meta-analysis showed that in healthy volunteers without a family history of depression no changes in mood occurred^14^. However, in persons at risk for depression, including healthy volunteers with a positive family history and remitted depressed patients, a decrease in mood did occur, with the effects being more prominent for remitted patients. Moreover, a deterioration of mood after ATD in remitted depressed patients predicted a higher chance to develop a new depressive episode^15^. In current depressed patients no mood response was observed^14^, probably because the disorder is already present^16^, and thus there is no risk for developing a depression.

The serotonergic system also plays a role in the pathogenesis of anxiety disorders^11,17^. A positron emission tomography study showed that compared to controls, patients with social anxiety disorder (SAD) had an overactive presynaptic serotonin system. Increased serotonin synthesis and serotonin transporter availability was observed in several brain regions^18^. Serotonin is also the mode of action of antidepressants, which are effective in treating anxiety disorders^19,20^. Details of the underlying mechanisms of the role of the serotonin system in anxiety disorders are still unknown. Given that ATD seems to reveal vulnerability in depressive disorders and the involvement of the serotonergic system in both disorders, ATD could potentially reveal vulnerability for developing anxiety disorders.

The first aim of this study was to systematically review the effects of ATD in healthy volunteers and patients with a remitted or current anxiety disorder on a change in reported levels of anxiety. Provided that ATD reveals vulnerability in anxiety disorders, it is expected, in line with the findings for ATD in depression^14^, that for healthy volunteers ATD will have little effect on anxiety, that ATD will increase anxiety in remitted patients, and that no effect will be found of ATD on anxiety in current patients.

A difference between depressive disorders and anxiety disorders is that a negative mood in depressive disorders seems more constantly present, whereas anxiety occurs predominantly when a patient is exposed to anxiety-provoking situations. Therefore, anxiety might need to be challenged. The second aim of this study is, thus, to review whether provoking anxiety by means of a challenge test modifies the anxious response after ATD. The challenge test is expected to increase anxiety independent of ATD. It is expected that using a challenge test after ATD will result in a more prominent anxious response.

## Methods

### Literature search

PubMed, Embase and PsychInfo were searched by an experienced librarian and SMES from inception to April 2019. The search string consisted of (combinations of) free text and keywords indicative for anxiety disorders and tryptophan depletion (search strategy in Appendix 1). Language was unrestricted. The search was extended by scanning the references of the included articles.

Inclusion criteria were: (1) Studies focused on healthy volunteers with or without anxiety symptoms, or patients with a remitted or current PD; agoraphobia (AG); SAD; or generalised anxiety disorder (GAD). Comorbidity was allowed. (2) ATD was conducted measuring anxiety symptoms pre- and post-depletion. (3) The studies had either a within-subject or a between-subject design, with an ATD and sham intervention arm. In a within-subject design participants served as their own control. Animal studies, articles not presenting original data, consisting of abstracts only, or written in languages other than English or Dutch were excluded.

In accordance with the PRISMA guidelines^21,22^, study selection was conducted by two independent reviewers (SMES and NMB). Firstly, studies were assessed for eligibility based on titles and abstracts. Secondly, both reviewers independently assessed the method section of selected articles. Discrepancies were resolved through discussion.

### Data extraction and analysis

For each article SMES and NMB independently extracted the following data: publication year, study design, presence and type of anxiety disorder, number of participants per intervention sequence, male/female ratio, used anxiety scales, outcomes on anxiety scales, use and type of challenge test and the corresponding outcomes. Discrepancies were resolved by referral to the original article. The primary outcome was the change in anxiety scores pre- and post-ATD compared to the sham intervention on the scales used in the original articles. The secondary outcome was the effect of a challenge test on anxiety scores.

### Quality assessment and publication bias

Quality of studies was assessed using the Cochrane Collaboration tool as a guideline^23^. Assessed aspects were related to demographic and clinical characteristics, how ATD was conducted, and, if applicable, how the challenge test was conducted. RCB did the initial scoring and this was double-checked by NMB. In case of disagreement, consensus was reached through discussion.

Within the context of this, systematic review studies were rated to be of good quality when they used a randomised double-blind design, ATD was well executed and reported, and the relevant outcome measures were reported. When these items were insufficiently reported, the study was rated to be of medium quality. Studies which did not report the pre- and post-ATD anxiety scores nor the difference scores were rated to be of poor quality.

### ATD

ATD is a technique to lower brain serotonin by reducing blood plasma tryptophan. In humans, most plasma tryptophan is bound to albumin, the remaining ‘free tryptophan’ can pass the blood–brain barrier into the central nervous system^13^. In the brain the tryptophan hydroxylase enzyme first converts tryptophan into 5-hydroxytryptophan (5-HTP), which is then decarboxylated into 5-hydroxytryptamine (serotonin or 5-HT). The serotonin synthesis rate depends on the amount of free tryptophan in the blood, the amount of tryptophan passing the blood–brain barrier and the activity of the tryptophan hydroxylase enzyme. Along with tryptophan, five other large neutral amino acids (LNAAs) compete to pass the blood–brain barrier^24^. Maximum brain tryptophan depletion is achieved by combining a low tryptophan diet and an overnight fast with a drinkable tryptophan-deficient protein load containing large amounts of LNAAs. In healthy volunteers, concentrations of plasma tryptophan were highly correlated with cerebrospinal fluid levels of tryptophan, which suggests that ATD affects the serotonin turnover in humans in the central nervous system^25,26^. Plasma tryptophan levels are lowest 5–7 h post-ingestion and hence, it is assumed that the effects of low levels of serotonin can then be studied^26,27^.

### Quality assessment of ATD

The ‘golden standard’ for ATD consists of a low tryptophan diet 24 h before the test day, preceded by an overnight fast, which is extended throughout the day. For ATD a 100 g load of 15 amino acids, including other LNAAs but without tryptophan is given. This procedure tends to reduce tryptophan levels in the blood with approximately 80% 5–7 h post-ingestion^26,27^ and is considered high quality ATD. Reduction of plasma tryptophan levels is determined with a blood test.

In the selected studies, the quality of ATD was determined based on the description of the plasma tryptophan levels (free or total [free + bound to albumin]) before and after depletion and on the ratio between plasma tryptophan and other LNAAs. The percentage of change was calculated based on the reported tryptophan levels or LNAA ratio. In this study, ATD was defined to be of sufficient quality when plasma tryptophan decreased by at least 50%. There is, however, still debate on when ATD can be considered to be of ‘good’ quality^28^.

## Results

### Literature search

**Fig. 1:**
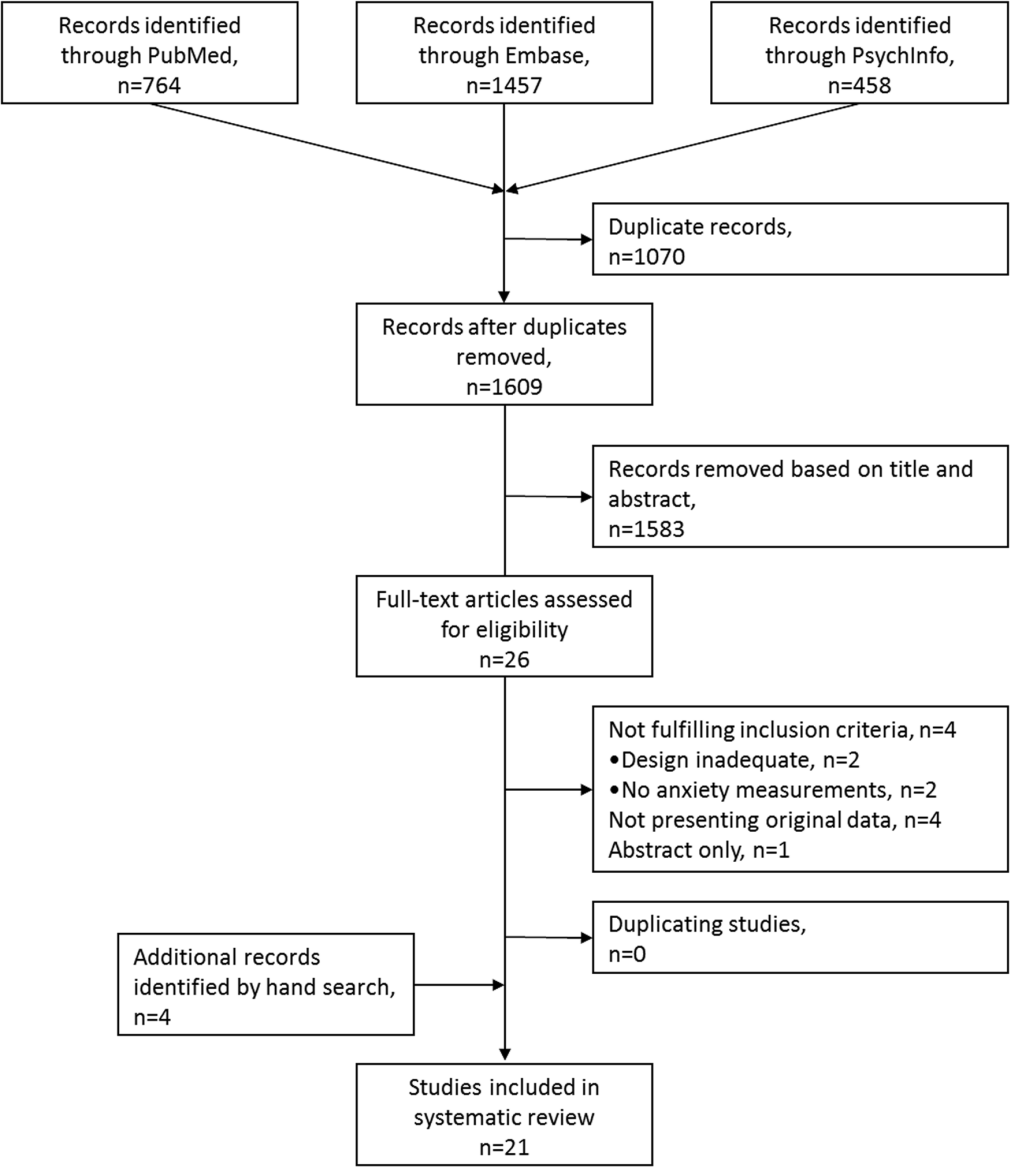
Flowchart of the literature search.

The search in PubMed, Embase and PsychInfo resulted in 1609 unique records; of these, 1583 were excluded based on their title and abstract, resulting in 26 records for full-text screening (Fig. 1). Based on the inclusion criteria, 17 unique studies were identified with anxiety as outcome measure^29–45^. Cross referencing of the included articles resulted in the additional inclusion of four articles^46–49^. This systematic review thus contains 21 studies.

### Study characteristics

Table 1 provides an overview of the characteristics of the 21 included studies. The studies were published between 1987 and 2012, and consisted of 16 within-subject designs and 5 between-subject designs. The sample size ranged from 8 to 80. Eleven studies investigated healthy volunteers, six remitted patients, two current patients and two both healthy volunteers and current patients. Investigated anxiety disorders were GAD (*n* = 1), SAD (*n* = 2) and PD (*n* = 8).

**Table 1:** Characteristics of included studies.

Reference	Design	Diagnosis	Sample size *N* (men)	Age Mean (SD)^a^	ATD^b^	Challenge	Result Anxiety scores (scale, scores)^c^	
							Effect of ATD alone compared to control condition or group, unless otherwise specified	Effect of ATD with challenge test compared to control condition of group
**Healthy controls**								
Smith et al. 1987 (47)	Rand., DB, Betw. SS	HC	80 (80)	^a^18–25	+	-	MAACL: - No sign. effect	
Benkelfat et al. 1994 (45)	Rand., DB, within SS	HC	39 (39) 20 FH + 19 FH-	FH + 24.1 (2.8) FH- 22.3 (3.4)	±	-	POMS anxiety: - Sign. effect (F[1,37] = 13.1, *p* < 0.001) STAI-S: - No sign. effect (F[1,36] = 3.0, *p* < 0.095)	
Park et al. 1994 (48)	DB, Within SS	HC	12 (12)	29; ^a^21–39	+	-	VAS anxiety: - No sign. effect	
Cleare and Bond 1995 (46)	DB, Betw. SS	HC	48 (48) 24 HTA 24 LTA	HTA: 32 (9) LTA: 33 (8)	+	Competitive reaction time task	MRS-anxiety/calmness subscale: - No sign. effect STAI-S:- No sign. effect	MRS-anxiety/calmness subscale:- No sign. effect STAI-S: - No sign. effect
Goddard et al. 1995 (41)	Rand., DB, Within SS	HC	11 (10)	27 (2)	±	Yohimbine	VAS anxiety:- No sign. effect (F[7,70]=0.91, p = 0.5) VAS nervousness: - No sign. effect (F[7,70]=1.43, p = 0.3)	VAS anxiety:- No sign. effect of yohimbine-ATD on anxiety compared to yohimbine-alone (F[7,70] = 0.96, *p* < 0.25) - Sign. effect of yohimbine-ATD on anxiety compared to placebo (F[7,63] = 2.11, *p* < 0.05) VAS nervousness: - Sign. effect of yohimbine-ATD on anxiety compared to yohimbine-alone (F[7,70] = 3.02, *p* < 0.005) - Sign. effect of yohimbine-ATD on anxiety compared to placebo (F[7,63] = 4.13, *p* < 0.0005)
Kent et al. 1996 (30)	Rand., Within SS	HC PD	HC: 7 (5) PD: 5 (3)	27.2 (3.96), range 24-34	+	CO_2_− 5%	-	API-IIIR: - No sign. effect 10 point anxiety scale: - No sign. effect 10 point apprehension scale: - No sign. effect Panic attacks: - No sign. effect
Koszycki et al. 1996 (31)	Rand., DB, Between SS	HC	40 (40)	ATD: 23.8 (0.8); Sham-depletion: 25.4 (1.0)	+	CCK-4	-	VAS anxiety:- No sign. effect VAS fear:- No sign. effect VAS nervousness: - No sign. effect PSS-IIIR: - No sign. effect Panic attacks: - No sign. effect (χ^2^ = 0.10, p = 0.75)
Klaassen et al. 1998 (32)	Rand., DB, Within SS	HC	15 (15)	29 (4)	+	CO_2_− 35%	STAI-S: - Sign. effect (*Z* = −2.02, df = 14, *p* = 0.04) POMS-tension: - No sign. effect VAS anxiety: - No sign. effect	VAS anxiety: - No sign. effect PSL-IIIR: - Sign. net increase (post-challenge minus pre-challenge) (*Z* = −1.96, df = 14, *P* = 0.05) Panic Attacks: - No sign. effect
Miller et al. 2000 (43)	Rand., DB, Within SS	HC PD	HC: 19 (11) PD: 20 (10)	HC: 29.1 (8.4) PD: 38.4 (9.9)	+	CO_2_− 5%	VAS anxiety: - No sign. effect VAS panic: - No sign. effect POMS: - No sign. effect STAI-S: - No sign. effect *Results PD see below*	VAS anxiety: - No sign. effect VAS panic: - No sign. effect POMS: - No sign. effect STAI-S: - No sign. effect API-IIIR: - No sign. effect (F[1,17] = 0.46, *p* = 0.508) *Results PD see below*
Monteiro-Dos-Santos et al. 2000 (33)	Rand., DB, Betw. SS	HC	29 (15) ATD: 15 (8) Control (7)	ATD: 27.9 (1.3) Control: 28.1 (0.9)	±	SPS	-	STAI-S: - Sign. effect in females for speech anxiety (F[1,9] = 8.93, *p* = 0.012), but not in males - VAMS-anxiety: No sign. effect
Shansis et al. 2000 (42)	Rand., DB, Within SS	HC	12 (12)	23.7: ^a^21–31	±	SPS	POMS: - No sign. effect VAMS: No sign. effect	VAMS: - - No sign. effect
Colasanti et al. 2011 (35)	Rand., DB, Within SS	HC	18 (10)	25 (5.5)	+	CO_2_− 35%	VAAS: - No sign. effect PSL-IV: - No sign. effect POMS: - No sign. effect	VAAS: - Sign. effect (F = 5.79, *p* < 0.05, df’s not reported) PSL-IV: - No sign. effect
Robinson et al. 2012 (53)	Rand., DB, Within SS	HC	20 (13)	25.1 (5.6)	+	Startle Paradigm	STAI-S: - No sign. effect (*p* > 0.1)	STAI-S: - No sign. effect (*p* > 0.1) Retrospective anxiety: - No sign. effect (*p* > 0.1)
**Remitted patients**								
Bell et al. 2002 (38)	Rand., DB, Within SS	PD remitted with SSRI >12 wks	14 (7)	40.6; ^a^ 21–65	+	Flumazenil	STAI-S: - No sign. effect PSI: - Sign. effect at 240 min (*t* = 2.946;df = 14; *p* < 0.05) VAS anxiety: - No sign. effect Panic attacks: - Sign. effect with 10/14 or with tighter PSI definition 7/14 panic attacks in ATD condition compared to 1/14 in control condition (*p* = 0.016)	STAI-S: - No sign. effect PSI: - No sign. effect VAS anxiety: - No sign. effect
Argyropoulos et al. 2004 (39)	Rand., DB, Within SS	SAD remitted on SSRI	14 (9)	39.9 (11.2), range 21–58	±	ABS	STAI-S: - Sign. effect (F[1,12] = 9.369, *p* = 0.01) VAS anxiety: - No sign. effect (*p* = 0.20)	STAI-S: - Sign. effect for autobiographical script (*z* = −2.28, *p* = 0.02) and neutral script (*z* = −2.2, *p* = 0.03), but not for verbal task (*z* = 1.49, *p* = 0.14) VAS anxiety: - Sign. effect for autobiographical script (*z* = −2.26, *p* = 0.02), but not for neutral script (*z* = −0.31, *p* = 0.75) or verbal task (*z* = −0.34, *p* = 0.73)
Davies et al. 2006 (36)	Rand., DB, Within SS	PD with - SSRI - CBT SAD with SSRI	27 (15) 14 7 6	39.2 (12.0)	+	Flumazenil ABS	STAI-S: - No sign. effect (*p* = 0.385) PSI - No sign. effect (*p* = 0.052)	STAI-S: - Sign. effect (*p* = 0.001) PSI: - Sign. effect (*p* = 0.013)
Tõru et al. 2006 (28)	Rand., DB, Within SS	PD remitted on SSRI >2 wks	18 (6)	34.5 (9.3)	+	CCK-4	VAS anxiety: - No sign. effect PSS-IV: - No sign. effect	VAS anxiety: - No sign. effect (*U* = 161.0, *p* = 0.99) PSS-IV: - No sign. effect (*p* = 0.70) Panic attacks: - No sign. effect (χ^2^ = 0.13, df = 1, *p* = 0.72)
Hood et al. 2010 (37)	Rand., DB, Within SS	GAD remitted on SSRI	11 (5)	37.5 (12.2)	+	Chronic CO_2_−7,5%		STAI-S: - No sign. effect VAS anxiety: - No sign. effect POMS: - - No sign. effect
Bell et al. 2011 (44)	Rand., DB, Within SS	PD remitted on CBT	9 (5)	33.2, range 20–43	+	Flumazenil	STAI-S: - No sign. effect PSI: - No sign. effect VAS anxiety: - No sign. effect	STAI-S: - Sign. effect (F[3,24] = 3.1, *p* = 0.04) PSI: - No sign. effect (F[3, 240] = 1.5, *p* = 0.240) VAS anxiety: - No sign. effect (F[1,24] = 3.9, *p* = 0.08) Panic attacks: - No sign. effect (*p* = 0.625)
**Patients**								
Goddard et al. 1994 (40)	Rand., DB, Within SS	PD (unmedicated)	8 (4)	42 (7)	+	-	VAS anxiety: - No sign. effect Panic attacks: - No sign. effect	
Kent et al. 1996 (30)	Rand., Within SS	HC PD	HC: 7 (5) PD: 5 (3)	27.2 (3.96), range 24–34	+	CO_2_− 5%	Results for patients were the same as for healthy volunteers (see above)	
Miller et al. 2000 (43)	Rand., DB, Within SS	HC PD	HC: 19 (11) PD: 20 (10)	HC: 29.1 (8.4) PD: 38.4 (9.9)	+	CO_2_− 5%	VAS anxiety: - No sign. effect VAS panic: - No sign. effect up to 240 min. - Sign. effect between 240–270 min (F[1,37] = 4.37, *p* = 0.043) POMS: - No sign. effect STAI-S:- No sign. effect	VAS anxiety: - No sign. effect (F[2,34] = 3.07, *p* = 0.060) VAS panic:- Sign. effect (F[2,34] = 6.14, *p* = 0.005) POMS:- No sign. effect STAI-S: - No sign. effect API:- Sign. effect (F[1,34] = 23.06, *p* < 0.001)
Schruers et al. 2000 (34)	Rand., BD, Betw. SS	PD	24 (9) Depletion: 12 (5) Control 12 (4)	Depletion: 43 (12) Control (37 (11)	+	CO_2_− 35%	PSL-IV: - No sign. effect VAAS: - No sign. effect	PSL-IV: - Sign. effect (*p* < 0.02) VAAS: - Sign. effect (*p* < 0.05) Panic attacks:- Sign. effect (*p* < 0.01)

*ABS* autobiographic script, *API* Acute Panic Inventory, *ATD* acute tryptophan depletion, *Betw. SS* between-subjects design, *CBT* cognitive behaviour therapy, *CCK-4* tetrapeptide central cholecystokinin receptor agonist, *CO2* carbon dioxide, *DB* double-blind, *FH* multigenerational family history of affective illness, *GAD* generalised anxiety disorder, *HC* healthy controls, *HTA* high trait aggression, *LTA* low trait aggression, *MAACL* Multiple Affect Adjective Checklist, *MRS* mood rating scale, *PD* panic disorder, *POMS* Profile of Mood States, *PSI* panic symptom inventory, *PSL* panic symptom list, *PSS* panic symptom scale, *Rand.* randomised, *SAD* social anxiety disorder, *sign.* significant, *SPS* simulated public speaking, *STAI-S* state part of State Trade Anxiety Inventory, *SSRI* selective serotonin reuptake inhibitor, *VAAS* visual analogue anxiety scale, *VAMS* Bond and Lader visual analogue mood scale, *VAS* visual analogue rating scale, *Within SS* Within subjects design.

^a^If the Mean or SD was not reported, the range is noted.

^b^ATD + : good depletion, ±: unclear level of depletion was adequate.

^c^Statistics only reported if described in original article.

Ten different challenge tests to provoke anxiety symptoms were used in 17 studies. Studies with healthy volunteers used tetrapeptide central cholecystokinin receptor agonist (CCK-4), 5–35% carbon dioxide (CO_2_), reaction time task, simulated public speaking (SPS), startle paradigm and yohimbine. Studies with PD patients used CCK-4, 5–35% CO_2_ and flumazenil. Both studies with SAD patients used an autobiographic script and the study with GAD patients used 7.5% CO_2_ for at least 12 min.

To assess anxiety 18 different outcome measures were used. The most commonly used scales were anxiety visual analogue scales (VAS-A) with scale end-points that varied between studies (*n* = 11), the state part of the State Trait Anxiety Inventory (STAI-S) (*n* = 11) and the Profile of Mood States (POMS) (*n* = 6). Due to the variety in included populations, the use of different challenge tests and the broad range of outcome measures the studies were considered too heterogeneous to be pooled for meta-analysis.

### Healthy volunteers

Thirteen studies were conducted in healthy volunteers (*n* = 350). Based on ten studies there is little evidence that ATD alone increased anxiety in healthy volunteers. Two studies reported a significant increase in anxiety following ATD, but the findings were inconsistent with the other anxiety measures used^41,46^. None of the other studies found a significant effect of ATD alone on anxiety^30,35–37,44,47–49^.

Ten studies used a challenge test. The effect of the challenge test alone on anxiety without ATD was determined by looking at the results of the sham depletion, unless the study reported the effects of the challenge test separately. The challenge test alone increased anxiety in six studies^35,40–42,44,47^, although most studies did not report whether the increase was statistically significant. In two studies the challenge test alone resulted in an increase on some anxiety measures, but not on others^30,37^. Two studies did not report the scores on the anxiety measures, the effect of the challenge test alone is thus unknown^36,39^.

The 5% CO_2_ challenge, reaction time task, yohimbine or CCK-4 did not increase anxiety following ATD^35,37,39,40,47^ compared to the challenge without ATD. Mixed results were found for the 35% CO_2_ challenge, SPS and startle paradigm^30,36,41,42,44^.

Studies in healthy volunteers are heterogeneous with respect to the number of outcome measures (*n* = 17) and challenge tests (*n* = 7) used. In healthy volunteers neither ATD alone nor ATD with a challenge test consistently increased anxiety compared to sham depletion or sham depletion with a challenge test, respectively.

### Remitted patients

Six studies were conducted in remitted patients (*n* = 93). Patients were treated with cognitive behavioural therapy (CBT) (*n* = 1)^38^, with either CBT or a selective serotonin reuptake inhibitor (SSRI) (*n* = 1)^45^, or with SSRIs (*n* = 4)^29,31–33^. Five studies reported the effects of ATD alone. In three studies there were no indications that ATD increased anxiety^29,38,45^, and two studies reported mixed results^32,33^, with significant results on some but not on other anxiety measures.

In all six studies the challenge test alone increased anxiety, although it is unknown whether this increase was statistically significant.

The results of the challenge test following ATD were mixed. There were no indications that 7.5% CO_2_ or CCK-4 increased anxiety in remitted GAD or PD patients^29,31^. Flumazenil was used to provoke panic symptoms in remitted PD patients with mixed results^32,38,45^: two studies used the same anxiety measures^32,38^ with three out of four measures providing no indications for increased anxiety and the significant measure differed between studies. In the third study flumazenil with ATD did consistently increase anxiety^45^. Results of this study are difficult to interpret, as both PD and SAD patients were included. They received flumazenil or an autobiographical script respectively and the results were not reported per challenge test. Another study using an autobiographical script found that the challenge did significantly increase anxiety after ATD in remitted SAD patients^33^.

Studies in patients with a remitted anxiety disorder are heterogeneous in terms of the included anxiety disorder (*n* = 3), the used outcome measures (*n* = 6) and challenge tests (*n* = 4). In remitted patients ATD alone did not consistently increase anxiety and the use of challenge tests provided mixed results.

### Current patients

Four studies were conducted in current PD patients (*n* = 57). Three studies reported the effects of ATD alone: in two studies anxiety was not increased^34,43^ and in one study results were mixed^37^.

Three studies used a challenge test. In two studies the challenge test alone increased anxiety^37,43^, although it is unknown whether this was statistically significant, and in one study the effect of the challenge alone is unknown as no anxiety scores were reported^39^.

Two studies used a 5% CO_2_ challenge test after ATD, in one study no effect on anxiety was found^39^ and in the other results were mixed^37^. A 35% CO_2_ challenge did result in a significant consistent increase in anxiety in current PD patients^43^.

Studies in current PD patients were heterogeneous in terms of outcome measures (*n* = 8). In current PD patients ATD alone did not increase anxiety. The use of a 35% CO_2_ challenge test after ATD did significantly increase anxiety, but this was not found for lower CO_2_ concentrations.

### Quality assessment and publication bias

In twelve studies a low tryptophan diet 24 h before ATD was included^29–31,33,34,36–40,42,46^, in three studies no diet was included^35,47,48^ and in five studies this was not reported^41,43–45,49^. The exact content of the diet varied between studies. Fifteen studies included an overnight fast^29,31–33,35–38,40,41,43,46–49^, but the duration of the fast varied. For the remaining studies it was unclear whether an overnight fast was required^30,34,39,42,44,45^. Blood tests showed that for total tryptophan the depletion ranged between 75.5–89.2%, for free tryptophan between 58.3–86.7% and the LNAA ratio between 61.35–92.7%. Studies measured the effect of ATD and the challenge test 4 to 7 h post-ingestion.

The quality assessment is reported in Table 2. In all studies except one^31^ bias may have affected the outcomes. First, in three studies it was unclear how diagnostic status was determined^47–49^ and in four studies no structured clinical interview was used^34,35,42,45^. Second, in two studies there were discrepancies between the reported and calculated percentages of change in plasma tryptophan levels^33,46^ and in three studies it was only reported that there was a significant difference between conditions, but the percentage change was not reported^35,36,42^. Third, in twelve studies^29,30,32–34,37,40,44,45,47–49^ there is a high risk for attrition bias as dropouts were not reported or the number of dropouts was high (>15%) without it being clear whether dropout was selective. Fourth, ten studies^30,32–36,38,40,43,46^ did not correct for multiple testing, though multiple comparisons were made using the anxiety and mood scales. Fifth, in eleven studies^29,32,34–39,41,44,47^ it was reported that there was a (non-)significant difference in anxiety and/or mood scores, but the pre-post test scores or difference scores were missing. Sixth, selection bias may be present in three studies^42,47,49^, as it was not clear whether participants were randomised. Finally, in one study^39^ there is a risk for detection bias as it is unclear if the study was conducted in a double-blind fashion. Overall, six studies were judged to be of ‘good’ quality because no major biases were present, five studies judged to be of ‘medium’ quality and ten studies of ‘poor’ quality.

**Table 2:** Quality assessment of included studies. Items are derived from Cochrane Collaboration Tool for bias assessment.

Reference	Population	Tryptophan depletion	Challenge test	Statistical analysis	Dropouts	Selective reporting and incomplete outcome data	Randomisation	Double blind fashion	Outcome assessor blind	Judgment^a^
**Healthy volunteers**										
Smith et al. 1987 (47)	High	Low	Not applicable	Low	High	Low	Low	Low	Low	Good
Benkelfat et al. 1994 (45)	Low	Medium	Not applicable	Medium	Medium	Low	Low	Low	Low	Medium
Park et al. 1994 (48)	High	Low	Not applicable	Low	High	Low	Medium	Low	Low	Medium
Cleare and Bond 1995 (46)	High	Low	Not applicable	Low	High	Medium	Medium	Low	Low	Poor
Goddard et al. 1995 (41)	Medium	Medium	Low	Medium	Low	Medium	Low	Low	Low	Poor
Kent et al. 1996 (30)	Low	Low	Low	Low	Medium	Medium	Low	Medium	Low	Poor
Koszycki et al. 1996 (31)	Low	Low	Low	Medium	High	Low	Low	Low	Low	Good
Klaassen et al. 1998 (32)	Low	Low	Low	Low	Low	Medium	Low	Low	Low	Poor
Miller et al. 2000 (43)	Low	Low	Low	Low	High	Medium	Low	Low	Low	Poor
Monteiro-dos-Santos et al. 2000 (33)	Medium	Medium	Low	Low	Low	Low	Medium	Low	Low	Medium
Shansis et al. 2000 (42)	Low	Medium	Low	Medium	Low	Medium	Low	Low	Low	Poor
Colasanti et al. 2011 (35)	Low	Low	Low	Low	High	Medium	Low	Low	Low	Poor
Robinson et al. 2012 (29)	Low	Low	Low	Medium	High	Low	Low	Low	Low	Good
**Remitted patients**										
Bell et al. 2002 (38)	Low	Low	Low	Medium	High	Medium	Low	Low	Low	Poor
Argyropoulos et al. 2004 (39)	Low	Medium	Low	Medium	High	Low	Low	Low	Low	Medium
Davies et al. 2006 (36)	Medium	Low	Low	Low	High	Low	Low	Low	Low	Good
Tõru et al. 2006 (28)	Low	Low	Medium	Low	High	Medium	Low	Low	Low	Poor
Hood et al. 2010 (37)	Low	Low	Low	Low	Low	Low	Low	Low	Low	Good
Bell et al. 2011 (44)	Low	Low	Low	Medium	Low	Medium	Low	Low	Low	Poor
**Current patients**										
Goddard et al. 1994 (40)	Medium	Low	Not applicable	Medium	High	Medium	Low	Low	Low	Poor
Kent et al. 1996 (30)	Please see under healthy volunteers									
Miller et al. 2000 (43)	Please see under healthy volunteers									
Schruers et al. 2000 (34)	Low	Low	Low	Medium	Low	Low	Low	Low	Low	Good

^a^Studies were judged to be of ‘good’ quality when using a double-blind randomised design, tryptophan depletion was well executed and reported, and the pre- and post ATD outcome measures were reported. In ‘medium’ quality studies one of the previous items was insufficiently reported. In ‘poor’ quality studies the pre- and post ATD scores nor the differences scores were reported.

## Discussion

This systematic review aimed to investigate whether, in line with depression, ATD could reveal vulnerability for developing an anxiety disorder. In addition, we examined whether challenging anxiety modifies anxiety after ATD. Only 21 studies could be identified. The included studies were mostly of limited quality and heterogeneous in terms of included populations, challenge tests and outcome measures. As in depression^14^, anxiety did not consistently increase after ATD alone in healthy volunteers and current patients. Against expectations, there was also no consistent anxious response in remitted patients. With a challenge test, anxiety did not consistently increase in healthy volunteers, which is in line with their limited vulnerability for onset of anxiety disorders. Contrasting our hypothesis, although at risk^6^, a challenge test after ATD did not provoke anxiety in remitted patients. Only in current PD patients a 35% CO_2_ test resulted in a consistent increase in anxiety on all measures after ATD^43^. This is possibly related to the suffocation alarm function PD patients being oversensitive. When challenged with CO_2_ PD patients have an increased brainstem response compared to controls and the brainstem response is positively correlated with physical panic attack symptoms^50^. To what extend this is related to the underlying pathology is questionable, since patients with bilateral amygdalae lesions also respond to a CO_2_ challenge with panic and fear^51^. Other studies in current PD patients used a 5% concentration of CO_2_^37,39^ and did not show this result. Currently there is little evidence that ATD can reveal vulnerability in anxiety disorders, which contrasts with the findings for depression.

In addition to dysregulations in the serotonin system^11^, several other factors also contribute to the pathogenesis of anxiety disorders, including genetic, psychological and social factors (e.g.^52–56^). Studies in this systematic review were generally not controlled for such factors, while these factors could affect the outcomes of the studies. Variability in these factors among participants might explain why no effect of ATD on anxiety was observed. The serotonin system also plays a key role in depressive disorders^11,17^. In research for depression the impact of such factors was shown. It was for example previously found that the serotonin transporter genotype modulates the response to ATD^57–59^. Patients with homozygous long allele variants of the serotonin transporter gene SLC6A4 were generally more likely to respond with a depressed mood to ATD than patients with a homozygous short allele of this gene^57–59^. However, most ATD studies in depression did not control for factors that could have affected the outcomes, but nevertheless did find an effect of ATD on mood. It is therefore surprising that we found no indications for ATD revealing vulnerability in anxiety, in particular in remitted patients. To increase our understanding, we are thus in need of high quality studies with large sample sizes which do control for other factors, like genetic factors, that could affect the response to ATD.

A potential explanation for the lack of findings in remitted anxiety disorders is Deakin and Graeff’s theory that serotonin mediates the response to aversive stimuli^60^. This theory assumes that the serotonin system is an anticipatory system to distal threats which influences the emotions fear and anxiety differentially: fear-related responses are inhibited from activating too early, while serotonin can facilitate anxiety responses to aversive stimuli^60^. ATD does not seem to result in a response in fear-related disorders^61^. Most anxiety disorders belong to the ‘fear’ spectrum, namely SAD, specific phobia, AP and PD, in which anxiety mostly increases in feared situations. This is in contrast to GAD which is, together with depressive disorders, classified on the ‘anxiety-misery’ spectrum^62^, in which anxiety or a negative mood is present most of the time. Given this subdivision, findings for GAD might be more similar to the findings for depressive disorders than for the fear-spectrum anxiety disorders. Only one small study included patients with remitted GAD (*n* = 11)^31^ and no significant effects were found. Given that of the remitted depressed patients 50–60% responds with a negative mood to ATD^63^, further investigation of GAD in larger populations may be of interest.

Alternatively, the timeframe for response to ATD for anxiety disorders might be later than for depressive disorders. This is congruent with the timing of the evaluation of the effect of antidepressant treatment, which according to the guidelines is for depressive disorders between 4 and 6 weeks and for anxiety disorders between 6 and 12 weeks^64–68^. This difference in response latency may suggest a different involvement of the serotonergic system in these disorders or different interactions with other neurotransmitter systems. Possibly the timing of the evaluation of response needs to be later for anxiety than for depression. Instead, an anxious response may take longer to develop, which may require longer tryptophan depletion than necessary for a mood response. This remains to be investigated.

In depression, studies do not use a challenge test^14^, while in this systematic review 17 of 21 studies in anxiety used such a test. An explanation could be that, in depression, a negative mood is present most of the time, while anxiety generally increases in specific situations. Even when a challenge test is used, results showed no consistent effect of ATD on anxiety.

The challenge test may however induce ceiling effects and as a result no additional effect of ATD can be detected. For example, in healthy volunteers in whom ATD was combined with a yohimbine challenge, their anxious response was significantly increased after ATD with yohimbine compared to placebo, but not in comparison to yohimbine alone^35^.

### Strengths and limitations

A strength of this study is its systematic approach summarising probably all published ATD studies including anxiety measures. This study provides an overview of the published ATD research in anxiety, providing insight in the gaps of the current knowledge. Findings should be interpreted in context of the following limitations.

First, the selected studies showed heterogeneity. Due to the inclusion of different populations, anxiety disorders, challenge tests and outcome measures, results are difficult to compare and meta-analysis is precluded. The variability in the execution of ATD could also lead to false-positive/negative results and hinders comparison between studies.

Second, many studies were of poor quality. Mostly because the scores on outcome measures of anxiety were not reported. Furthermore, it could not always be determined how the diagnostic status was assessed, whether dropouts were selective and whether randomisation had taken place.

Third, several studies found a significant increase on one anxiety measurement^32,33,37,38,41,42,44,45^, but results were often not corrected for multiple testing which could result in false positive findings. Furthermore, the measures that showed an effect differed between studies and different results have been observed between studies using the same measures in similar populations with a similar challenge test.

Fourth, the timing of the measures varied between studies. For example, post-ingestion and challenge test measures were taken 4–7 h post-ingestion. This could have affected the results, as the peak of tryptophan depletion is 5–7 h post-ingestion^26,27^.

Fifth, the sample sizes of the included studies were small, possibly resulting in underpowered studies and therefore lack of significant results. Although studies in depressive disorders had similar sample sizes, for anxiety fewer studies have been conducted (21 studies up to April 2019 versus 76 studies up to 2007). Patterns may thus not be elucidated due to the relatively low number of studies and the observed heterogeneity.

Sixth, results are mostly described on group level. Similar to depressive disorders it could be that only specific groups of participants respond with anxiety (e.g.^14,57^) and that this remains unobserved in the group effects.

Seventh, it remains to be determined which challenge tests are suitable for inducing anxiety after ATD. Challenge tests have been used which correspond with the characteristics of the disorder. It needs investigating to what extend these challenge tests result in ceiling effects and which challenge tests are most suitable to be used in healthy volunteers.

### Conclusion

This systematic review showed that ATD research in anxiety disorders is heterogeneous, often of poor quality, and few studies have been conducted. Unlike in depressive disorders, ATD does not seem to be able to function as a marker of vulnerability for developing anxiety disorders. In anxiety disorders there is, thus, a need for good quality studies with large samples, which do control for other factors that could affect the response to ATD, before more definite conclusion can be drawn about lack of suitability of ATD as a marker for vulnerability in anxiety disorders.

## Supplementary information

Appendix 1 Search Strategy
