# Supplementary material for: Effects of tryptophan depletion on anxiety, a systematic review
Source: Transl Psychiatry. 2021 Feb 11;11:118. doi: 10.1038/s41398-021-01219-8 (PMC7878770; doi:10.1038/s41398-021-01219-8)
Supplement: Supplementary file 1 — Appendix 1 Search Strategy [file 41398_2021_1219_MOESM1_ESM.docx]

**Appendix 1 Search strategy PubMed**

Search (("Tryptophan"[Mesh] OR "Tryptophan Hydroxylase"[Mesh] OR tryptophan* [tiab] OR 5-hydroxytryptophan* [tiab] OR hydroxytrytophan* [tiab] OR 5-hydroxy-tryptophan*[tiab] OR 5-HTP [tiab] OR oxitriptan* [tiab] OR serotonin depletion* [tiab])) AND ("Anxiety Disorders"[Mesh] OR “Anxiety” [Mesh] OR anxiet* [tiab] OR anxious* [tiab] OR agoraphobi* [tiab] OR panic* [tiab] OR phobi* [tiab] OR nervousnes* [tiab])

This search strategy has been translated for additional searches in both Embase and PsychInfo.
